# Juvenile idiopathische Arthritis – persistierender oligoartikulärer Subtyp: Konsensusbasierte Handlungsempfehlungen im Rahmen der ProKind Rheuma Initiative

**DOI:** 10.1007/s00393-025-01762-5

**Published:** 2025-12-12

**Authors:** Joachim Peitz, Gerd Horneff, Anna Raab, Hanna Winowski, Sandra Hansmann, Klaus Tenbrock

**Affiliations:** 1 Kinderrheumazentrum, Sankt Augustin, Zentrum für Allgemein Pädiatrie und Neonatologie, Asklepios Klinik Sankt Augustin, Sankt Augustin, Deutschland; 2 Klinik für pädiatrische Pneumologie, Allergologie und Neonatologie, Abteilung Pädiatrische Rheumatologie, Medizinische Hochschule Hannover, Hannover, Deutschland; 3 Klinik für Kinder- und Jugendrheumatologie, St. Josef Stift Sendenhorst, Sendenhorst, Deutschland; 4 Klinik für Kinder- und Jugendmedizin, Abteilung III, Universitätsklinikum Tübingen, Tübingen, Deutschland; 5 Klinik für Kinder- und Jugendmedizin, Translationale Pädiatrische Rheumatologie und Immunologie, Klinikum der RWTH Aachen, Pauwelsstr 30, 52074 Aachen, Deutschland; 6 Klinik für Kinderheilkunde, Pädiatrische Rheumatologie, Inselspital Universität Bern, Bern, Schweiz

**Keywords:** Treat to target, Kindliches Rheuma, Intraartikuläre Injektionen, Methotrexat, Off-label-Therapie, Treat-to-target management, Pediatric rheumatology, Intra-articular injections, Methotrexate, Off-label use

## Abstract

Für die oligoartikuläre Form der juvenilen idiopathischen Arthritis (JIA) wurden im Rahmen eines konsentierten Prozesses Protokolle entwickelt, welche die Klassifikation, Überwachung und Therapie betreffen (ProKind Rheuma). Durch die Autorengruppe wurden 23 Statements formuliert und diese im Rahmen eines Online-Survey zur Beteiligung unter den ärztlichen Mitgliedern der Gesellschaft für Kinder- und Jugendrheumatologie (GKJR) zirkuliert. An der Beantwortung des Surveys nahmen insgesamt 80 der insgesamt 124 Kinder- und JugendrheumatologInnen teil; dies entspricht einem Anteil von knapp 65 % der zu dem Zeitpunkt aktiven Kinder- und JugendrheumatologInnen. In einem abschließenden Online-Meeting wurden Anmerkungen aus der Umfrage in die Statements eingearbeitet und dann von der Autorengruppe konsentiert. Für die neu auftretende oligoartikuläre JIA wurden 20 Statements und ein zusammenfassendes Konsensus-Therapieprotokoll entwickelt; diese sollen die Behandlung der persistierenden oligoartikulären JIA optimieren.

## Abstract (en)

Protocols concerning classification, monitoring and treatment were developed for the oligoarticular form of juvenile idiopathic arthritis (JIA) as part of a consensus process. The group of authors formulated 23 statements and circulated them in an online survey to medical members of the Society for Paediatric and Adolescent Rheumatology (GKJR). A total of 80 of the 124 paediatric and adolescent rheumatologists took part in the survey, which corresponds to just under 65% of the paediatric and adolescent rheumatologists who were active at the time. In a final online meeting, comments from the survey were incorporated into the statements and then agreed upon by the group of authors. For newly occurring oligoarticular JIA, 20 statements and a summary consensus treatment protocol were developed to optimise the treatment of persistent oligoarticular JIA.

Die oligoartikuläre juvenile idiopathische Arthritis (JIA) ist die am häufigsten diagnostizierte Kategorie der chronischen Arthritis bei Kindern in Europa und Nordamerika. Sie macht 50–80 % der Fälle chronischer Arthritis bei Kindern aus [13], dennoch gibt es keine evidenzbasierten Leitlinien für die Behandlung.

Im Rahmen des Projektes Protokolle in der Kinderrheumatologie (ProKind) wurden, beginnend mit der polyartikulären JIA, Protokolle zur Diagnostik, Klassifikation, Überwachung und Therapie erstellt nach einem zuvor konsentierten Prozess [5]. Das hier dargestellte Pro-Kind-Protokoll für das erste Behandlungsjahr der oligoartikulären JIA (ILAR Kategorie persistierende Oligoarthritis; [9]) wurde nach diesem Prozess erarbeitet und soll als Anleitung zur Indikation, Durchführung und Überwachung der Therapie im klinischen Alltag dienen. Alle ProKind-Protokolle orientieren sich inhaltlich an der vorhandenen Evidenz, den aktuellen Zulassungssituationen von Medikamenten und am aktuellen Vorgehen im klinischen Alltag sowie den bisher publizierten Leitlinien, die nicht durch diese ProKind-Protokolle ersetzt werden. Vielmehr sollen sie der Harmonisierung der Diagnostik sowie einer einheitlichen Befunddokumentation und einer nachvollziehbaren Therapie dienen, um die Wirksamkeit unterschiedlicher Therapie bewerten zu können und dadurch die Behandlung von Kindern mit JIA optimieren zu können.

Die bereits in den Leitlinien und auch in den anderen Protokollen zur JIA formulierten Behandlungsgrundsätze gelten auch hier: „Das Hauptziel der Therapie ist die rasche und effektive Entzündungsbehandlung mit entsprechender Schmerzbekämpfung, die Kontrolle der Grunderkrankung und ggf. die Remissionsinduktion, die Vermeidung von körperlicher Behinderung durch Gelenkkontrakturen, Gelenkdestruktionen, Wachstumsstörungen in den betroffenen Gelenken mit der Folge von Fehlstellungen, Erhalt der Sehkraft, Vermeidung der Schädigung innerer Organe, Unterstützung bei psychosozialer Belastung des Patienten und der Familie, Gewährleistung einer weitgehend störungsfreien somatischen und psychosozialen Entwicklung der Kinder- und Jugendlichen“. Die Voraussetzungen für eine erfolgreiche Therapie der JIA ist eine frühzeitige Diagnosestellung und Zuweisung der Patienten an Ärzte und Ärztinnen mit Kompetenz und Erfahrung in der Behandlung der JIA [8]. Die psychosoziale Betreuung, ein Bewusstsein für psychosoziale Komorbiditäten, nichtmedikamentöse Therapiekonzepte, insbesondere Physiotherapie und eine gut vorbereitete, koordinierte und geplante Transition, sind verankerte Prinzipien in der letzten S2k-Leitlinie „Therapie der juvenilen idiopathischen Arthritis“ [8].

## Definition der oligoartikulären JIA

Die Definition und Klassifikation der JIA erfolgen aktuell nach den Kriterien der International League of Associations for Rheumatology (ILAR; [9, 11]). Die Diagnose der JIA steht für eine chronische, zumindest 6 Wochen persistierende Arthritis mit einem Erkrankungsbeginn vor dem 16. Lebensjahr nach Ausschluss anderer Ursachen. Somit liegt eine chronische Arthritis unklarer Genese vor. Die Subklassifikation der JIA erfolgt in 6 Diagnosekategorien und letztlich erst nach Ablauf der ersten 6 Erkrankungsmonate in Abhängigkeit von der Anzahl betroffener Gelenke und extraartikulärer Manifestationen. Bei der persistierenden Oligoarthritis dürfen in den ersten 6 Monaten maximal 4 Gelenke betroffen sein. Ausschlusskriterien für die persistierende oligoartikuläre JIA sind z. B. HLA-B27-Positivität bei Jungen mit Erkrankungsalter über 6 Jahre, das Vorliegen einer Psoriasis oder auch der wiederholte Nachweis von Rheumafaktoren [2, 14]. Bei der „extended“ oligoartikulären JIA sind im Verlauf mehr als 4 Gelenke betroffen. Für diese Erkrankung sind dieselben Medikamente wie für die polyartikuläre JIA zugelassen, so dass hierzu auf die Protokolle für die polyartikuläre JIA zurückgegriffen werden sollte [5].

Differenzialdiagnostisch auszuschließen sind bei der oligoartikulären JIA vor allem infektassoziierte Arthritiden, insbesondere die septische Arthritis oder die Lyme-Arthritis. Eine reaktive Arthritis mit schmerzhaften und rasch auftretender Arthritis sowie schlechtem Ansprechen auf nichtsteroidale Antirheumatika kann ebenfalls eine Differenzialdiagnose sein.

Die Reihe der weiteren Differenzialdiagnosen umfasst nichtentzündliche Ursachen, vor allem Traumafolgen, Stoffwechselerkrankungen (z. B. Zöliakie oder Glykogenosen), hereditäre hämatologische Erkrankungen (Hämophilie, Thalassämie), Überbelastungszustände, aber auch in seltenen Fällen onkologische Erkrankungen, wie Knochentumoren oder Leukämien.

Bei zusätzlichen Symptomen wie Fieber, Hautausschlag, anderen Organmanifestationen sollte differenzialdiagnostisch auch an eine Kollagenose oder ein Fiebersyndrom, z. B. familiäres Mittelmeerfieber (FMF) gedacht werden.

Bisher liegen keine Protokolle zu Diagnostik, Klassifikation, Überwachung und Therapie der persistierenden oligoartikulären JIA vor. Das Ziel der Pro-Kind-Projektgruppe war es daher, die derzeitigen Praxismuster hinsichtlich der oligoartikulären JIA zu beschreiben und ein konsensbasiertes Vorgehen zu Diagnostik, Überwachung und Therapie im ersten Erkrankungsjahr zu erstellen.

## Methoden

### Prozessbeschreibung zur Entwicklung der Protokolle

Die Erstellung der konsentierten Therapieprotokolle der Gesellschaft für Kinder- und Jugendrheumatologie (GKJR) – in diesem Falle der persistierenden oligoartikulären JIA im Rahmen des Projektes ProKind – folgte einem mehrstufigen Prozess. Initial wurden im Jahr 2015 bei einem Vorbereitungstreffen, zu dem alle Mitglieder der GKJR per Email eingeladen waren, neun verschiedene Diagnosen als *besonders dringlich zu behandeln* definiert [4, 5].

1. Polyartikuläre JIA
2. Persistierende oligoartikuläre JIA
3. Enthesitis-assoziierte Arthritis (EAA-JIA)
4. Systemische JIA
5. JIA-Uveitis
6. Familiäres Mittelmeerfieber
7. CASPS/TRAPS/HIDS
8. Systemischer Lupus erythematodes
9. Juvenile Dermatomyositis

**Abb. 1:**
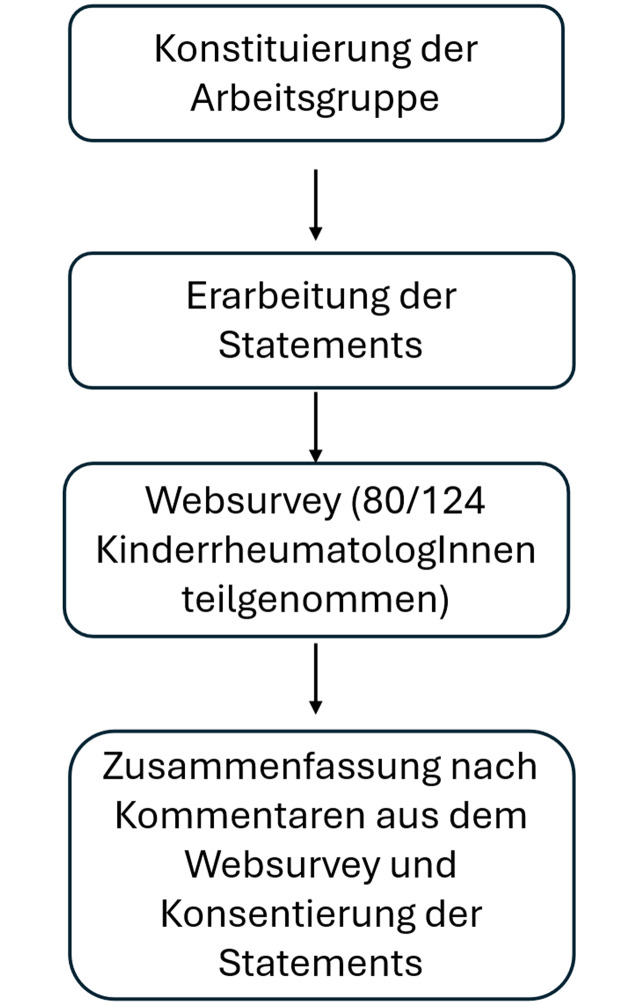
Arbeitsablauf des Konsentierungsprozesses für die persistierende Oligoarthritis

Nach dem Vorbereitungstreffen wurde von der GKJR an alle Mitglieder eine Einladung zur aktiven Beteiligung verschickt. Im Rahmen des Vorbereitungstreffens wurde auch ein mögliches Vorgehen zur Konsentierung von Protokollen vorgeschlagen (Abb. 1):

1. Erstellung eines Entwurfes durch mindestens einen Teil der Arbeitsgruppe
2. Verteilung des Entwurfes an die übrigen Teilnehmer der Arbeitsgruppe
3. Konsentierung innerhalb der Arbeitsgruppe (Konsens bei Zustimmung von mindestens 80 % der Teilnehmenden) mittels Telefonkonferenz
4. Aufforderung zur Konsentierung des ausgearbeiteten Entwurfes an alle GKJR-Mitglieder im Rahmen eines WebSurvey (auch hier Konsens bei Zustimmung von mindestens 80 % der Teilnehmer)
5. Ergebniskonsentierung innerhalb der Arbeitsgruppe und ggf. – insbesondere bei größeren Veränderungen – Vorlage an alle GKJR-Mitglieder im Rahmen eines zweiten WebSurvey
6. Verabschiedung der Protokolle bei Face-to-Face-Online-Meeting
7. Autorisierung durch den Vorstand der GKJR und Zustimmung zur Publikation (Web- und/oder Print)

Die Arbeitsgruppe für das Pro-Kind-Protokoll für die persistierende oligoartikuläre JIA bestand aus 6 Mitgliedern, die sich zur Arbeit an diesem Thema bereit erklärt hatten und welche die Autoren dieses Artikels sind. Es wurden insgesamt 23 Empfehlungen anhand der vorhandenen Literatur zur Klassifikation, Überwachung und Therapie innerhalb der Arbeitsgruppe in drei Telefonkonferenzen erarbeitet. Wie im oben beschriebenen Prozess vorgesehen, wurden alle Empfehlungen mit einer Mehrheit von mindestens 80 % innerhalb der Arbeitsgruppe verabschiedet. Anschließend wurden alle Empfehlungen im Rahmen eines WebSurvey an alle GKJR-Mitglieder zur Konsentierung verschickt.

## Ergebnisse

Die Zustimmungshäufigkeit der 80 Teilnehmer des WebSurvey sind in Tab. 1 wiedergegeben. Nach Rücklauf der Ergebnisse der Web-Umfrage wurden die Antworten analysiert, und insgesamt wurde ein sehr hoher Grad der Zustimmung festgestellt. Insgesamt wurde eine Zustimmung zwischen 87,3 und 100 % erreicht. Hiermit wären formal alle Aussagen als konsentiert angenommen gewesen.

**Tab. 1:** Zustimmungsverhalten aus dem Survey der ärztlichen GKJR-Mitglieder

Aussage	Zustimmung		Diskussion	Ablehnung	
	Ich stimme zu	Ich stimme zu mit Vorschlag	Ich stimme nicht ohne Veränderung zu	Ich stimme nicht zu	Gesamt-Zustimmung in %
1	0,97	0,03	0	0	100
2	0,56	0,38	0,05	0,01	93,5
3	0,97	0,01	0,01	0	98,7
4	0,68	0,22	0,08	0,01	90,79
5	0,80	0,11	0,07	0,03	90,79
6	0,85	0,08	0,07	0	93,05
7	0,93	0,03	0,03	0,01	95,94
8	0,61	0,31	0,07	0,01	91,89
9	0,82	0,14	0,03	0,01	98,94
10	0,96	0,03	0	0,01	98,65
11	0,75	0,19	0,06	0	94,44
12	0,85	0,11	0,03	0,01	95,78
13	0,89	0,1	0,01	0	98,61
14	0,86	0,1	0,04	0	95,78
15	0,82	0,1	0,03	0,06	91,66
16	0,83	0,1	0,05	0,01	93,05
17	0,77	0,1	0,08	0,04	*87,32*
18	0,87	0,08	0,04	0	95,83
19	0,87	0,08	0,03	0,01	95,77
20	0,93	0,06	0	0,01	98,62
21	0,87	0,07	0,01	0,04	94,36
22	0,83	0,08	0,06	0,03	91,55
23	0,66	0,25	0,07	0,01	91,55

Die ersten beiden Antwortmöglichkeiten waren so formuliert, dass sie als Zustimmung ohne Bedingung gewertet wurden. Hierbei gab es jedoch bei der zweiten Antwort die Möglichkeit, einen Vorschlag zur Veränderung anzubringen. Bei den Antwortmöglichkeiten 3 und 4 wurde der Aussage nicht zugestimmt, wobei im Rahmen der dritten Antwort die Zustimmung von einer Änderung der Aussage abhängig gemacht wurde. Die Auswahl der ersten beiden Antwortmöglichkeiten wurde insgesamt als Zustimmung definiert.

Wiederholt geäußerte Veränderungsvorschläge aus der zweiten und dritten Antwortmöglichkeit wurden zusammengestellt. Vorgeschlagene Veränderungen wurden in der Arbeitsgruppe diskutiert, und insofern diese die Aussagen veränderten, auch protokolliert. Dies fand im Rahmen eines Face-to-Face-Meetings statt. Als Ergebnis des Meetings der Arbeitsgruppe wurden dabei je 2 von 6 themenverwandten Statements zusammengefasst, so dass insgesamt 20 Statements konsentiert wurden.

Die zu den Nummern korrespondierenden Aussagen der endgültigen Fassung sind im Folgenden formuliert.

## Statements zu Diagnostik, Monitoring und Therapie

Die nachfolgenden Statements zu Diagnostik, Monitoring und Therapie sollen für eine gesicherte persistierende oligoartikuläre JIA gelten. *Übergeordnete Statements zur Diagnose* sind in Tab. 2 aufgeführt, *Statements zu Diagnostik und Monitoring* in Tab. 3, *allgemeine Therapie-Statements* in Tab. 4. Um die Diagnose einer oligoartikulären JIA stellen zu können, muss dieser eine mindestens 6 Wochen andauernde Arthritis in weniger als 4 Gelenken vorausgegangen sein.

**Tab. 2:** Übergeordnete Statements zur Diagnose

*Statement 1: *Das Konsensustherapieprotokoll soll für die folgende nach der ILAR-definierten JIA Kategorie gelten (Einschlusskriterien): oligoartikulärer Verlauf persistent. Das Konsensustherapieprotokoll soll nicht gelten für (Ausschlusskriterien): jede andere Form (systemische JIA, Psoriasisarthritis, enthesitisassoziierte (EAA-)JIA, Rheumafaktor-positive oder -negative polyartikuläre JIA, „extended“ oligoartikuläre JIA)
*Statement 2:* Die minimale erforderliche Basisdiagnostik für die Diagnose und die Differenzialdiagnostik soll einschließen: Blutbild inklusive Differenzialblutbild, BSG, CRP, ASAT, ALAT, GGT, Kreatinin, CK, LDH, AP, Urinstatus, ANA, HLA-B27, Zöliakie-Serologie. Bei Hinweisen auf eine vorausgegangene Infektion oder bei gastrointestinalen Symptomen sollte ggf. eine weiterführende zielgerichtete Diagnostik erfolgen (z. B. Borrelienserologie, Mykoplasmen, Yersinien, weitere reaktive Erreger bzw. Calprotectin im Stuhl). (A)

Anmerkungen zu Statement 1 und 2:

A: Statement 2 wurde aus den Statements 2 und 4 nach Anmerkungen aus dem WebSurvey zusammengefasst:

WebSurvey Statement 2: Die minimale erforderliche Basisdiagnostik für die Diagnose und die Differenzialdiagnostik soll einschließen: Blutbild inklusive Differenzialblutbild, Blutsenkungsgeschwindigkeit (BSG), C‑reaktives Protein (CRP), Aspartat-Aminotransferase (ASAT), Alanin-Aminotransferase (ALAT), Gamma-Glutamyltransferase (GGT), Kreatinin, Harnsäure, Kreatinkinase (CK), Laktatdehydrogenase (LDH), Alkalische Phosphatase (AP), Urinstatus, Rheumafaktor (RF), HLA-B27, antinukleäre Antikörper (ANA), Immunglobulin (Ig) G, IgA, M, Borrelienserologie

WebSurvey Statement 4: Bei Hinweisen auf eine vorausgegangene Infektion oder bei gastrointestinalen Symptomen sollte eine weiterführende zielgerichtete Diagnostik erfolgen (Antistreptolysin [ASL], Mykoplasmen, Yersinien bzw. *Helicobacter-pylori-*Antigen und Calprotectin im Stuhl, Zöliakie-Antikörper im Serum).

**Tab. 3:** Statements zu Diagnostik und Monitoring

*Statement 3: *Bei jedem Patienten sollte zeitnah, idealerweise innerhalb von 2 Wochen, der Ausschluss einer Uveitis erfolgen. Bei allen Kindern mit einer oligoartikulären JIA sollen im weiteren Verlauf regelmäßige augenärztliche Untersuchungen zum Ausschluss einer Uveitis erfolgen, initial in 3‑monatlichen Abständen, entsprechend der hierfür veröffentlichten AMWF-Leitlinie (Registernummer 045-012). (B)
*Statement 4:* Vor Beginn einer immunsuppressiven Therapie sollen bei unsicherem Impfstatus Impfantikörper (Varizellen-Zoster-Virus, Masern, Hepatitis B) bestimmt werden sowie eine Vervollständigung des Impfstatus angestrebt werden
*Statement 5: *Die bildgebende Diagnostik soll eine sonographische Untersuchung zumindest aller klinisch befallenen Gelenke enthalten. Eine weiterführende Bildgebung sollte bei unklaren Fällen zur differenzialdiagnostischen Abgrenzung oder bei Verdacht auf eine Kiefergelenkbeteiligung oder bei Halswirbelsäulenbefall erfolgen, z. B. Röntgenbild, Magnetresonanztomographie
*Statement 6: *Zur Erfassung der Krankheitsaktivität und der Funktionseinschränkung kann der Juvenile Arthritis Disease Activity Score-10 (JADAS-10) bzw. der Clinical JADAS-10 und der Childhood Health Assessment Questionnaire (CHAQ) genutzt werden. (C)
*Statement 7: *Definition von Sicherheitsparametern je nach Aktivitätsstatus und Therapie. Bei Kontrolluntersuchungen sollen erfolgen: Blutbild inklusive Differenzialblutbild, Blutsenkungsgeschwindigkeit (BSG), C‑reaktives Protein (CRP), Kreatinin, Alanin-Aminotransferase (ALAT), Gamma-Glutamyltransferase (GGT), Laktatdehydrogenase (LDH), Urinstatus
*Statement 8: *Klinische Kontrolluntersuchungen im ersten Behandlungsjahr sollen in Intervallen spätestens alle 4–6 Wochen bis zum Eintritt einer Besserung erfolgen, dann alle 3 Monate
*Statement 9: *Definition der Ziele der medikamentösen Therapie für das erste Behandlungsjahr: Eine JADAS-inaktive Erkrankung (JADAS-10 ≤ 1,4) ist das eigentliche Ziel, eine JADAS-MDA (minimale Krankheitsaktivität, JADAS-10 1,4 bis ≤ 4) ist ein „akzeptierbares“ Ziel, ein JADAS-10 > 4 entspricht einer nichtakzeptierbaren Krankheitsaktivität. Die Verhinderung von Gelenkschäden ist ein weiteres Therapieziel

Anmerkungen zu den Statements 3 bis 9:

B: Statement 3 und 21 zur Uveitis wurden zusammengefasst:

WebSurvey Statement 3: Bei jedem Patienten sollte zeitnah, idealerweise innerhalb von 2 Wochen, der Ausschluss einer Uveitis erfolgen. WebSurvey Statement 21: Bei allen Kindern mit einer oligoartikulären JIA sollen im Verlauf regelmäßige augenärztliche Untersuchungen zum Ausschluss einer Uveitis erfolgen, initial in 3‑monatlichen Abständen, entsprechend der hierfür veröffentlichten AMWF-Leitlinie (Registernummer 045-012).

C: zum Zeitpunkt der Konsensuskonferenz waren die neuen JADAS-Kriterien noch nicht veröffentlicht. Diese wurden jetzt aktualisiert in das Statement mit aufgenommen [14]. Der JADAS-10 von ≤ 1,4 entspricht einer JADAS-inaktiven Erkrankung, ein JADAS-10 von 1,5 bis ≤ 4 entspricht einer minimalen Krankheitsaktivität, ein JADAS-10 von 4,1 bis ≤ 13 entspricht einer moderaten Krankheitsaktivität und ein JADAS-10 > 13 einer hohen Krankheitsaktivität. Für den cJADAS sind die Grenzwerte ≤ 1,1; 1,2–4; 4,1–12 und > 12.

**Tab. 4:** Allgemeine Therapiestatements

*Statement 10: *Eine symptomatische Therapie mit nichtsteroidalen Antiphlogistika soll bei entsprechender Symptomatik mit den gebräuchlichen Dosierungen, möglichst im Rahmen der Zulassung, erfolgen (Tab. 5)
*Statement 11: *Der alleinige Einsatz von nichtsteroidalen Antirheumatika (NSAR) kann ausreichend sein, so bei innerhalb weniger Wochen oder Monate selbstlimitierender Erkrankung. Bei über mehr als 4 Wochen anhaltender aktiver Arthritis mit Schmerzen und/oder beeinträchtigender Bewegungseinschränkung ist eine alleinige Therapie mit NSAR inadäquat. (D)
*Statement 12: *Eine Indikation zur intraartikulären Therapie mit Kortikosteroiden kann bei jeder aktiven Arthritis bestehen. Diese kann einen initialen Therapiebaustein darstellen oder im Verlauf zusätzlich zu anderen Therapiemaßnahmen erfolgen. Eine Wiederholung der intraartikulären Therapie mit Kortikosteroiden sollte bei Therapieversagen in der Regel nicht vor 3 Monaten nach letzter Injektion erfolgen. In Einzelfällen kann bei ausbleibender Wirkung eine zeitnahe Wiederholung der Injektion erwogen werden. (E)
*Statement 13: *Triamcinolonhexacetonid (TH) ist anderen Präparaten vorzuziehen. Dosis für TH 0,5–1 mg/kg Körpergewicht in große Gelenke max. 40 mg/Gelenk (Knie, Hüfte, Schulter), bis zu 0,5 mg/kgKG in mittelgroße Gelenke (Hand- Sprung‑, Ellenbogengelenke) max. 20 mg/Gelenk und bis max. 2 mg pro kleinem Gelenk (Finger, Zehen). (F)
*Statement 14: *Eine Indikation zur systemischen Therapie mit Methotrexat (MTX) (10–15 mg/m^2^KOF 1‑mal/Woche) kann bei jeder aktiven Arthritis bestehen. Diese kann einen initialen Therapiebaustein darstellen oder im Verlauf zusätzlich zu anderen Therapiemaßnahmen erfolgen, jedoch sollte hierbei überprüft werden, ob die Überführung in ein anderes ProKind-Protokoll notwendig erscheint. Eine Zulassung für die oligoartikuläre JIA besteht für MTX nicht. (G)
*Statement 15: *Eine Indikation zur systemischen Therapie mit Sulfasalazin (50 mg/kgKG max. 2 g/d) kann bei jeder aktiven Arthritis – nach Ausreizen der zugelassenen Therapie – bestehen, insbesondere bei Nachweis von HLA-B27. Diese kann einen initialen Therapiebaustein darstellen oder im Verlauf zusätzlich zu anderen Therapiemaßnahmen erfolgen. Auf jeden Fall sollte eine Überprüfung der Diagnose erfolgen und ggf. dann entsprechend die Überführung in ein anderes ProKind-Protokoll erwogen werden. Eine Zulassung für die oligoartikuläre JIA besteht für Sulfasalazin nicht. (H)
*Statement 16: *Ein Therapieversagen kann als Fehlen einer Besserung des JADAS definiert werden. Des Weiteren kann ein Therapieversagen festgestellt werden, wenn nach einer Therapiedauer von zumindest 6 Monaten nicht die Grenze für eine akzeptable Krankheitsaktivität unterschritten wurde
*Statement 17: *Des Weiteren kann ein Therapieversagen festgestellt werden, wenn nach einer Therapiedauer von zumindest 12 Monaten nicht die Grenze für eine minimale Krankheitsaktivität (JADAS-10 < 4) unterschritten wurde
*Statement 18: *Bei unzureichendem Ansprechen/Therapieversagen auf eine ausreichend ausgereizte Therapie mit intraartikulären Steroiden und NSAR kann eine Kombinationstherapie aus MTX und intraartikulärer Therapie mit Kortikosteroiden sinnvoll sein
*Statement 19: *Bei unzureichendem Ansprechen/Therapieversagen kann eine Erweiterung der Therapie mit Biologika oder „small molecules“ (Tumornekrosefaktor[TNF]-Blockade, CTLA4-Blockade, IL-6-Blockade, Januskinase[JAK]-Inhibitoren) in Erwägung gezogen werden. Für die oligoartikuläre JIA stellt dies einen „off-label use“ dar. Lediglich für die Uveitis ist Adalimumab zugelassen. Es sollte hierbei überprüft werden, ob die Überführung in ein anderes ProKind-Protokoll notwendig erscheint. (I)
*Statement 20: *Im Falle einer Remission unter Therapie sollte bei systemischer Therapie (MTX) die Medikation für mindestens 12 Monate beibehalten werden, im Einzelfall kann eine frühere Reduktion der Therapie sinnvoll sein

Anmerkungen zu den Statements 10 bis 20:

D: Zum Zeitpunkt der Diagnosestellung und Inkrafttreten dieses Statements besteht bereits eine aktive Arthritis seit 6 Wochen. Im Fall einer bis dahin unzureichenden Therapie mit NSAR, oder wenn zum Zeitpunkt der Diagnose eine Besserung eingetreten ist, kann eine alleinige Therapie mit NSAR über weitere 4 Wochen ausreichend sein. Bei darüberhinausgehender aktiver Arthritis sollte die Therapie jedoch erweitert werden.

E: Statement 12 wurde aus den Statements 13 und 15 des WebSurveys nach Anmerkungen zusammengefasst.

WebSurvey Statement 13: Eine Indikation zur intraartikulären Therapie mit Kortikosteroiden kann bei jeder aktiven Arthritis bestehen. Diese kann einen initialen Therapiebaustein darstellen oder im Verlauf zusätzlich zu anderen Therapiemaßnahmen erfolgen. Die Therapie lässt sich in mehrmonatigen Intervallen wiederholen.

WebSurvey Statement 15: Eine Wiederholung der intraartikulären Therapie mit Kortikosteroiden sollte bei Therapieversagen in der Regel nicht vor 3 Monaten nach letzter Injektion erfolgen.

F: [3, 16]

G: [1, 12]

H: [15]

I: Ein entsprechendes Protokoll wäre polyartikuläre JIA oder EAA [5, 6] Zum Zeitpunkt des Konsensus waren JAK-Inhibitoren auch für die polyartikuläre JIA noch nicht zugelassen, inzwischen besteht eine Zulassung für bestimmte JIA-Kategorien [10]. Die Autoren haben diese jetzt ergänzt.

Eine Empfehlung zu einem Off-label-Einsatz wird ausdrücklich nicht ausgesprochen. Jedoch kann eine Off-label-Therapie nach Einschätzung des behandelnden Arztes indiziert sein und ist möglich, wenn eine schwere gesundheitliche Beeinträchtigung oder mit Schmerzen verbundenes Leiden mangels therapeutischer Alternativen nicht wirksam behandelt werden kann. Zusätzlich müssen Forschungsergebnisse vorliegen, die erwarten lassen, dass das Arzneimittel für die betreffende Indikation zugelassen werden könnte (Tab. 5).

**Tab. 5:** Dosierung der nichtsteroidalen Antiphlogistika in der Therapie der oligoartikulären juveniler idiopathischer Arthritis (JIA)

Medikation	Tagesdosis	Bemerkung
Naproxen	10–15 mg/kgKG in 2 ED	Altersgrenze > 1 Jahr, Saftformulierung verfügbar
Ibuprofen	30–40 mg/kgKG in 3–4 ED	Zulassung im Kindesalter ab Alter 6 Monate, Saftformulierung verfügbar
Indometacin	2–3 mg/kgKG in 3–4 ED	Zulassung im Kindesalter ab Alter 2 Jahre, Saftformulierung verfügbar
Diclofenac	2–3 mg/kgKG in 2–3 ED	Zulassung im Kindesalter ab Alter 9 Jahre
Meloxicam	0,25–0,375 mg/kgKG in 1 ED	(Keine Zulassung für JIA, Zulassung ab 16 Jahre für rheumatoide Arthritis, ankylosierende Spondylitis und Arthrose)
Celecoxib	6–12 mg/kgKG in 2 ED	(Keine Zulassung für Kinder- und Jugendliche in Deutschland, Zulassung in den USA für Kinder ab 2 Jahren)

**Abb. 2:**
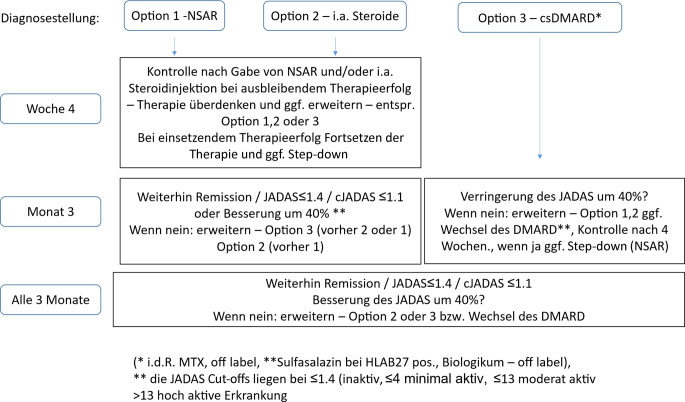
Konsensus-Therapieprotokoll zur oligoartikulären juvenilen idiopathischen Arthritis (ohne Uveitis): Insgesamt stehen 3 gleichwertige Therapieoptionen zur Wahl, die je nach Schwere des Befundes (wie eingeschränkt ist der Patient) sowie Präferenzen der Familie und des Therapiezentrums Anwendung finden. Sie können einzeln oder in Kombination verwendet werden. Außer bei einem Therapiebeginn mit einem krankheitsmodifizierenden antirheumatischen Arzneimittel (DMARD; Option 3) mit verzögertem Wirkeintritt, erscheint ein initialer Kontrollabstand von 4 Wochen zur Beurteilung des Therapieansprechens sinnvoll. Bei allen Therapieoptionen sollten regelmäßige Kontrollen alle 3 Monate erfolgen. Glukokortikoide in systemischer Applikation können bei hoher Krankheitsaktivität eingesetzt werden. Ein langfristiger Einsatz soll wegen unerwünschter Wirkungen und der Verfügbarkeit anderer Therapieformen nicht erfolgen (s. AWMF Leitlinie Juvenile idiopathische Arthritis (JIA), Ooomen et al.). Das Ziel ist eine Remission der Erkrankung nach spätestens 12 Monaten. Bei Entwicklung zu einer „extended“ oligoartikulären JIA wird der Wechsel in das Protokoll für die polyartikuläre JIA empfohlen. *DMARD* krankheitsmodifizierende antirheumatische Arzneimittel, *NSAR* nichtsteroidale Antirheumatika, *MTX* Methotrexat

Die Statements zur Therapie wurden von der Arbeitsgruppe zu einem Therapiealgorithmus zusammengefasst (Abb. 2).

## Diskussion

Für die persistierende oligoartikuläre JIA sind dies die ersten Protokolle zu Diagnostik, Klassifikation, Überwachung und Therapie. Mittels einer zuvor konsentierten Methode wurden initial 23 Empfehlungen erarbeitet und nach dem Survey zu insgesamt 20 Statements zusammengefasst und konsentiert. Die hier dargestellten Konsensusempfehlungen sind dabei explizit keine umfassende Leitlinie, sondern dienen primär der Harmonisierung von Diagnostik, Therapie und Monitoring der Erkrankung. Die Idee dieses ProKind-Prozesses ist, dass diese Harmonisierung zu einer Verbesserung der Behandlung im Sinne eines Treat-to-Target mit einer Verbesserung des Outcome führt. Limitiert werden diese Handlungsempfehlungen zudem vom Fehlen randomisierter klinischer Studien mit Ausnahme der Studie von Ravelli et al. [12] und der damit fehlenden Evidenz für die Wirksamkeit auch der verschiedenen Medikamente, die in der Off-label-Therapie genutzt werden. Das Verständnis der Pathogenese und die zunehmenden Therapieoptionen haben auch die Ziele bei der Behandlung chronisch-entzündlich-rheumatischer Gelenkerkrankungen im Kindesalter beeinflusst [17].

Heute sind eine geringe Krankheitsaktivität und nach Möglichkeit eine inaktive Erkrankung ein realistisches Therapieziel [2, 14]. Um dies für möglichst viele Patienten erreichbar zu machen, sind die vorliegenden Therapieprotokolle geeignet. Für die polyartikuläre JIA wurde dies in einer Treat-to-target-Studie bereits gezeigt [7].

Die Auswertung von Daten der mehr als 350 eingeschlossenen oligoartikulären Patienten im vom Gemeinsamen Bundesausschuss (GBA) geförderten Projekt ProKind Rheuma („treat to target“) unter der Leitung des Letztautors dieses Artikels legen dies ebenfalls nahe (Publikation in Vorbereitung). In dem Projekt werden Anwendung und Outcome des Treat-to-target-Ansatzes überprüft. Die Autoren dieses Konsensusartikels hoffen auf eine breite Durchdringung der Protokolle und des Treat-to-target-Ansatzes im klinischen Alltag.

## Fazit für die Praxis

- Im Rahmen des Projektes ProKind wurden nach einem konsentierten Prozess Protokolle zur Diagnostik, Klassifikation, Überwachung und Therapie der juvenilen idiopathischen Arthritis (JIA) erstellt.
- Das hier dargestellte Protokoll bietet eine Orientierung und Anleitung zur Indikation, Durchführung und Überwachung der Therapie im klinischen Alltag.
- Die Konsensusempfehlungen sollen in erster Linie der Harmonisierung von Diagnostik, Therapie und Monitoring der Erkrankung dienen und damit langfristig im Sinne eines Treat-to-target-Ansatzes zu einer Verbesserung der Behandlungsergebnisse führen.
- Therapieziele sind eine geringe Krankheitsaktivität und eine inaktive Erkrankung.
